# Construction and Analysis of Molecular Association Network by Combining Behavior Representation and Node Attributes

**DOI:** 10.3389/fgene.2019.01106

**Published:** 2019-11-07

**Authors:** Hai-Cheng Yi, Zhu-Hong You, Zhen-Hao Guo

**Affiliations:** ^1^Xinjiang Technical Institute of Physics and Chemistry, Chinese Academy of Sciences, Urumqi, China; ^2^University of Chinese Academy of Sciences, Beijing, China

**Keywords:** data analysis, network biology, machine learning, association prediction, graph embedding, miRNA-disease association

## Abstract

A key aim of post-genomic biomedical research is to systematically understand and model complex biomolecular activities based on a systematic perspective. Biomolecular interactions are widespread and interrelated, multiple biomolecules coordinate to sustain life activities, any disturbance of these complex connections can lead to abnormal of life activities or complex diseases. However, many existing researches usually only focus on individual intermolecular interactions. In this work, we revealed, constructed, and analyzed a large-scale molecular association network of multiple biomolecules in human by integrating associations among lncRNAs, miRNAs, proteins, drugs, and diseases, in which various associations are interconnected and any type of associations can be predicted. We propose Molecular Association Network (MAN)–High-Order Proximity preserved Embedding (HOPE), a novel network representation learning based method to fully exploit latent feature of biomolecules to accurately predict associations between molecules. More specifically, network representation learning algorithm HOPE was applied to learn behavior feature of nodes in the association network. Attribute features of nodes were also adopted. Then, a machine learning model CatBoost was trained to predict potential association between any nodes. The performance of our method was evaluated under five-fold cross validation. A case study to predict miRNA-disease associations was also conducted to verify the prediction capability. MAN-HOPE achieves high accuracy of 93.3% and area under the receiver operating characteristic curve of 0.9793. The experimental results demonstrate the novelty of our systematic understanding of the intermolecular associations, and enable systematic exploration of the landscape of molecular interactions that shape specialized cellular functions.

## Introduction

One key issue in the systems biology and genomics research is how different biomolecules interact with another to bring about the appropriate cellular activities (Barabási and Oltvai, 2004). There are various types of biomolecules in human that work together to achieve specific functions. And these intermolecular interactions are diverse, including protein-protein interactions (You et al., 2017), lncRNA-protein interactions (Yi et al., 2018), miRNA-lncRNA associations (Huang et al., 2017; Huang Z.-A et al., 2018), miRNA-disease associations (Chen et al., 2018a), miRNA-protein interactions (Dweep and Gretz, 2015), lncRNA-disease associations (Chen, 2015), protein-disease associations (Lee et al., 2012; Wang et al., 2018), drug-protein (target) associations (Chan and You, 2016; Li et al., 2017), drug-disease associations (Dumbreck et al., 2015; Wang et al., 2018; Zhang et al., 2018a) and so on. Many existing studies mainly focus on individual intermolecular interactions as mentioned above. When predicting the associations or interactions between two molecules, they generally only use the property information of the two kind of molecules themselves, such as the sequence or structure information of RNA or protein, the chemical structure information of the drug compound, and the semantic characterization information of the disease. This way, association patterns between nodes are lost. In fact, the interaction between these molecules is interconnected. For example, a protein may interact with lncRNA, but also interacts with another miRNA. In a magnified view, the connection between multiple biomolecules in a living organism is mutually exclusive. They should be considered in a comprehensive manner, analyzed, and modeled as a whole.

Interaction between two biomolecules alone has been thoroughly studied in the past few decades and has accumulated a lot of valuable data. A few researchers have noticed the connectivity of biomolecular interactions, but their use is very limited to a small number of two or three molecular elements. Alaimo et al. (2014) proposed a tripartite network by associating ncRNA with disease through its targets (genes) based on recommended system technique. Sun et al. (2015) comprehensively analyzed the network characteristics of disease genes and drug targets for five types disease, including cancer, metabolic disease, nervous system disease, immune system disease, and cardiovascular disease. Liu et al. (2017) constructed a heterogeneous network by combining the miRNA similarity which is calculated by miRNA-lncRNA associations and miRNA-target gene associations, and the disease similarity which is computed by the semantic similarity and functional similarity of disease. And the Random Walk is extended to predict miRNA-disease associations in the heterogeneous network. Ding et al. (Ding et al., 2018) presented a lncRNA-gene-disease tripartite graph to predict lncRNA-disease associations by connecting lncRNA-disease associations and gene-disease associations. Their approach is well characterized the heterogeneity of the associations between coding-noncoding genes-disease. Chen et al. (2018b) indicated a heterogeneous label propagation model to predict miRNA-disease association on the multi-network of miRNA, disease, and lncRNA. Vinayagam et al. (2016)Controllability analyzed the role of different protein nodes in the context of human disease and drug targets. These pioneer studies inspired that the interaction of biomolecules in human is diverse and synergistic, requiring systematic modeling and understanding of them.

From the previous studies, it also can be found that network, also known as graph, is an important data form to represent complex molecules associations (as links) between multiple biomolecules (as nodes). It is widely employed in various biomedical tasks, e.g. drug repositioning using drug-disease interactions network (Gottlieb et al., 2011), clinical decisions making through disease symptom network (Rotmensch et al., 2017) and identifying lncRNA functions based on lncRNA-protein interactions graph (Zhang et al., 2018b). In order to analyze these network data, network representation learning has been developed for many years, and many methods have been proposed. High-Order Proximity preserved Embedding (HOPE) (Ou et al., 2016) is a network embedding method based on matrix factorization. Its goal is to learn a low dimensional embedding of each node in a network, which can preserve network structure and node behavior information. Moreover, network representation learning can be used as a feature extraction technique to exploit discriminative features for entities such as diseases and microbes that have no direct physical or chemical information e.g. sequence and chemical structure for downstream specific machine learning tasks.

In *Human*, multiple biomolecules coordinate to sustain life activities. One disease is rarely a consequence of an abnormality in an isolated molecular interaction but reflected perturbations of the whole intermolecular interaction network (Barabási et al., 2010). Due to the functional interdependence between molecular components in human (Greene et al., 2015). We considered various types of biomolecules as entities (nodes), and the associations between them as links (edges), revealed and defined a comprehensive Molecular Association Network (MAN), which including most widely associations between miRNA, lncRNA, circRNA, protein (gene, mRNA), drug, microbe, and disease. It is also inspired by previous studies of bipartite or tripartite networks between biomolecules.

**Figure 1:**
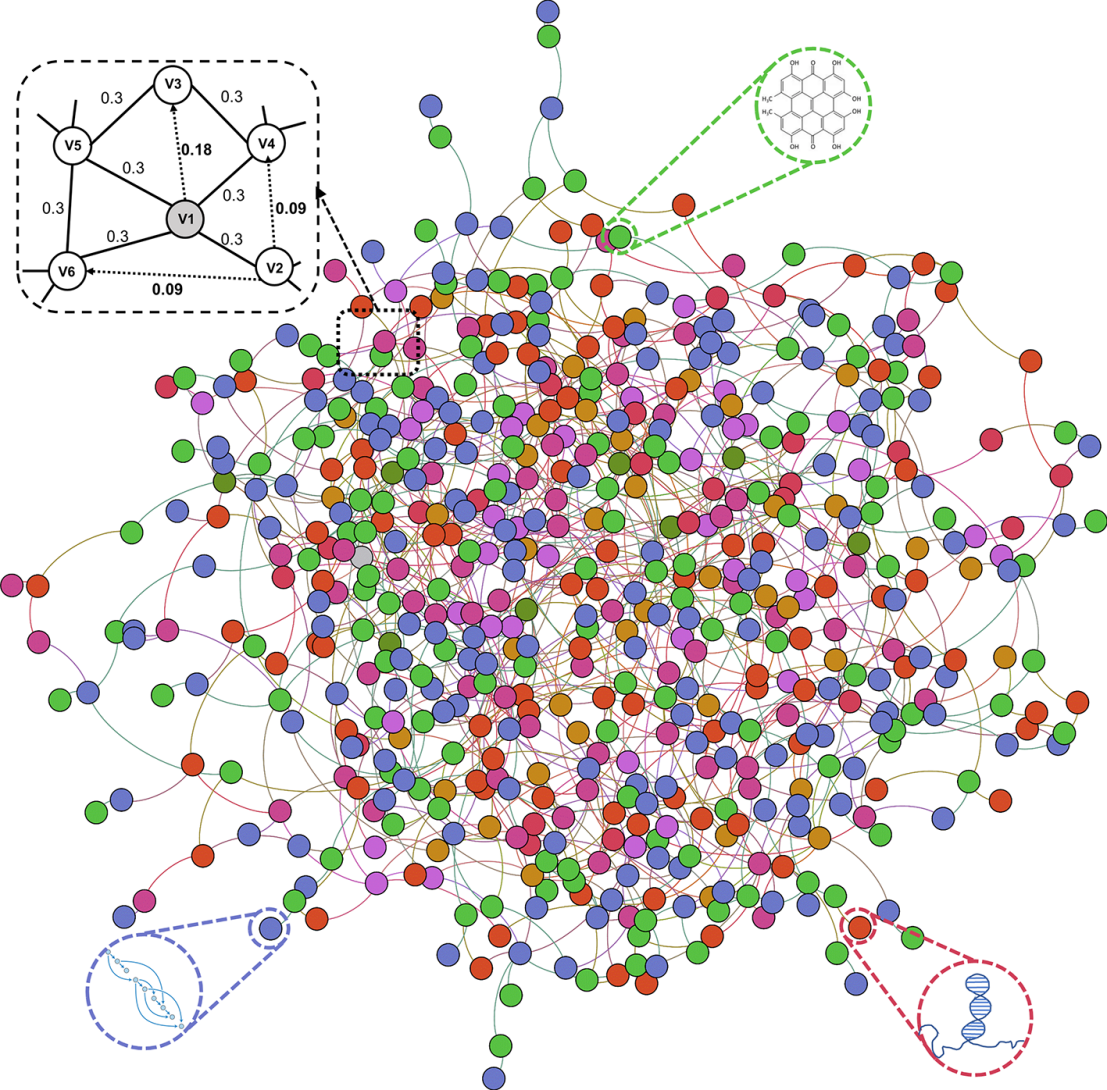
The workflow of MAN-HOPE.

In this work, we construct and systematically analyze the comprehensive molecular associations network in *Human*. We propose MAN-HOPE, a novel network-based representation learning framework to fully exploit latent embedding of biomolecules to accurately predict any presently unknown interactions between nodes. The workflow of MAN-HOPE is shown in **Figure 1**. More specifically, nine types of molecular associations, 105,546 links were systematically integrated to construct a MAN. The network representation learning model HOPE was employed to learn network embedding feature of each node in the MAN. Then, a Gradient Boosting Decision Trees (GBDT) model implemented by CatBoost (Prokhorenkova et al., 2018) was trained to predict the link (association) between nodes. To evaluate the prediction performance of our method, five-fold cross validation was executed. In order to prove the validity of the feature, we compared the effects of network behavior embedding features and traditional attribute features under same experimental conditions. Furthermore, we conducted a case study using our model to predict miRNA-disease associations in the MAN. Experimental results demonstrate the novelty of our systematic understanding of the interconnected network of intermolecular associations, and the role of MAN-HOPE in predicting any potential association between biomolecules.

## Materials and Methodology

### Construction of Molecular Association Network

**Figure 2:**
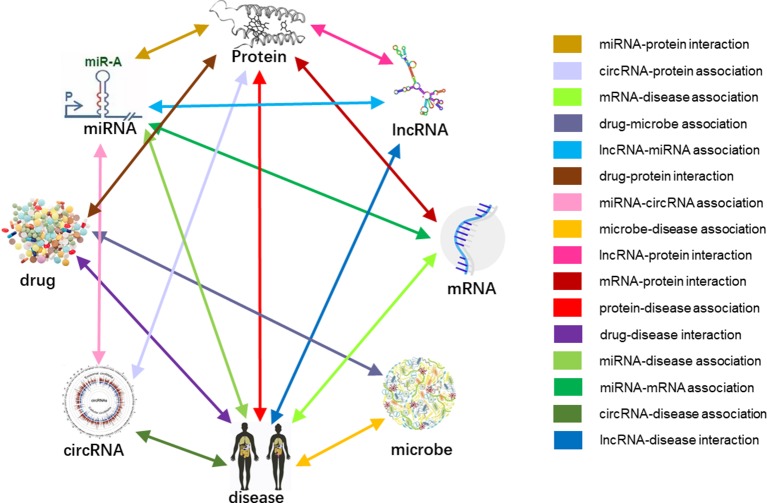
Construction of Molecular Association Network (MAN).

**Figure 3:**
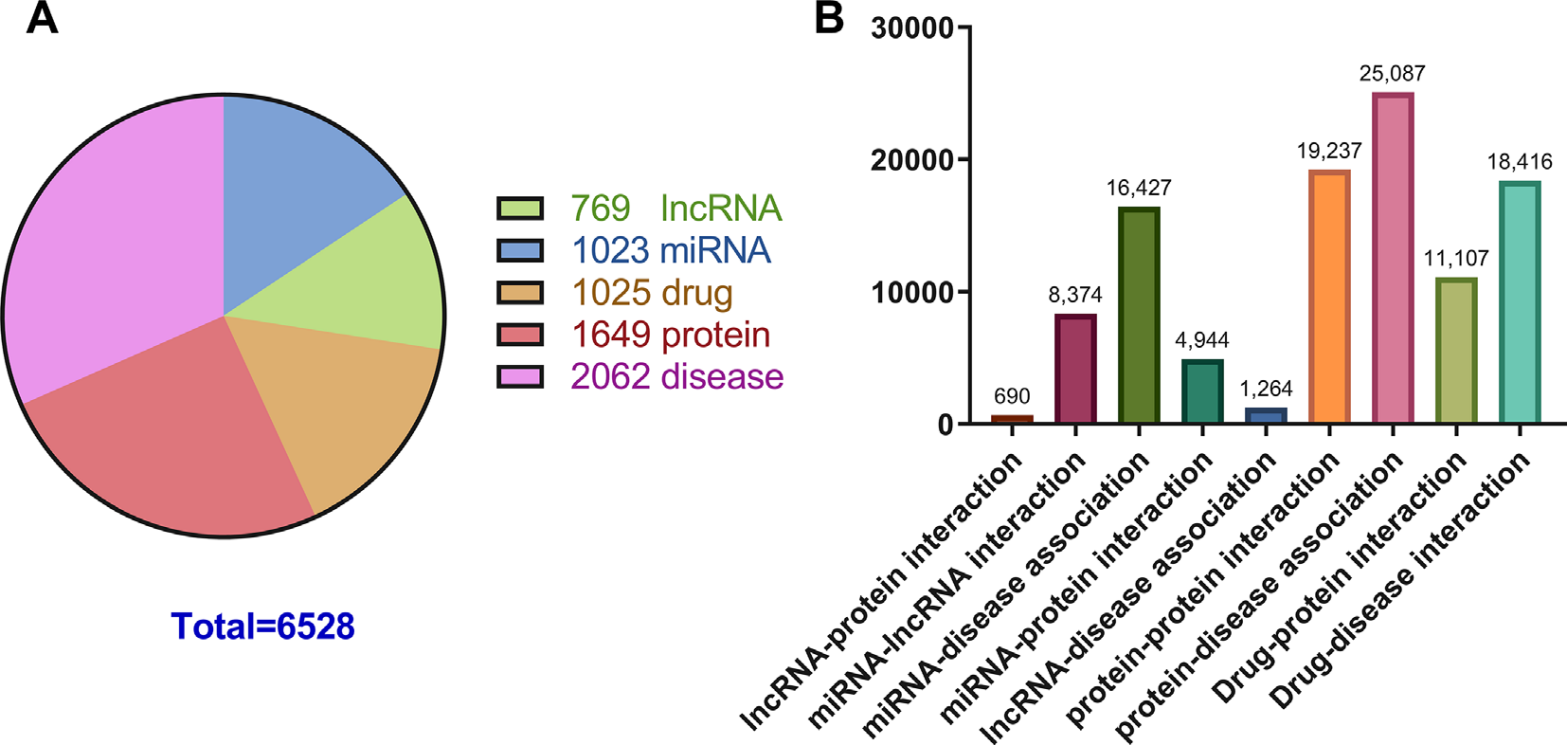
The distribution of molecular types and associations in the Molecular Association Network (MAN). **(A)** the distribution of lncRNA, miRNA, drug, protein, and disease nodes; **(B)** the type and amount details of molecular associations.

Previous studies on the interactions of two molecules provide plenty of beneficial data. We collect extensive data on the experimentally validated interactions between two molecules in human. The name of each type of entity is unified under one unified naming scheme to connect different interactions of the same molecules. Fully isolated pairs are removed. Schematic diagram of the MAN is shown in **Figure 2**. Finally, we obtained a MAN of 105,546 links, including 8,374 miRNA-lncRNA interactions from lncRNASNP2 (Miao et al., 2017), 16,427 miRNA-disease associations from HMDD (Huang Z et al., 2018), 4,944 miRNA-protein interactions from miRTarBase (Chou et al., 2017), 690 lncRNA-protein interactions from LncRNA2Target (Cheng et al., 2018), 25,087 protein-disease associations from DisGeNET (Piñero et al., 2016), 19,237 protein-protein interactions from STRING (Szklarczyk et al., 2016), 1,264 lncRNA-disease associations from LncRNADisease (Chen et al., 2012) and lncRNASNP2 (Miao et al., 2017), 18,416 drug-disease interactions from CTD (Davis et al., 2018), and 11,107 drug-protein (target) interactions from DrugBank (Wishart et al., 2017). The distribution of node types and interaction types in the MAN network is shown in **Figure 3** as follows.

### High-Order Proximity Preserved Embedding

In order to obtain efficient feature of nodes in large-scale network, the network representation learning model HOPE is employed to exploit network embedding of nodes in MAN. The HOPE algorithm characterizes two different representations for each node. Its main goal is to preserve the asymmetry information in the original network. HOPE constructs different asymmetric relation matrices and then uses the SVD algorithm for matrix decomposition to obtain the network representation of the node. For a given graph G = <V, E>, *V* represents the vertex set and *E* stand for the directed edge set. *S* is a high-order proximity matrix. *Z* = [*Z_s_*,*Z_t_*] is the embedding matrix. Compared with the Grape Factorization method, the HOPE model considers high-order similarity by introducing $S-Z_{s}Z_{2F}^{Tt}$ the Katz Index (Katz, 1953), Rooted PageRank (Song et al., 2009), Common Neighbors (Lorrain and White, 1971) and Adamic-Adar (Adamic and Adar, 2003) score and other similarity indicators were tried. And then, the similarity matrix is decomposed into

(1) $$S=M_{g}^{-1}M_{l}$$

Because the $M_{g}^{-1}$ and *M_l_* are both sparse matrices, so the use of SVD can have higher operating efficiency.

### Node Attributes

Each node in the MAN can be defined not only the network embedding, but also the attribute of themselves. In this work, for node with sequence information, the k-mer frequency was applied to exploit their attribute feature. For drug, Morgan fingerprints that represent their chemical structure are used as attribute features. For disease, we use a Medical Subject Headings (MeSH) descriptor describing the phenotype of the disease to construct a directed acyclic graph (DAG) to calculate disease similarity, using this measure of similarity as the attribute of the disease.

For sequence of miRNA, lncRNA, we use the 3-mer frequency to encode its sequence, from AAA to UUU, there is 4^3^ possible combinations of nucleic acid residues (A, C, G, U). For a given sequence, slide from left to right four residues as a sliding window, one residue one step, we can obtain the composition information of a sequence, and then, we normalize the feature vector according to the sequence length.

For protein, the processing of protein sequences is slightly different. The 20 amino acids are first divided into four groups according to the polarity of the side chain, which is inspired by existing protein study (Shen et al., 2007), including (Ala, Val, Leu, Ile, Met, Phe, Trp, Pro), (Gly, Ser, Thr, Cys, Asn, Gln, Tyr), (Arg, Lys, His), and (Asp, Glu). Then we can use the same 3-mer frequency mentioned above to process the protein sequence.

For drug, the chemical structure is represented by Simplified Molecular Input Line Entry Specification (Weininger, 1988), then we calculate corresponding Morgan fingerprints for each compound.

For disease, we use DAG to represent each disease based on MeSH descriptor. DAG(*D*) = (*D*, *N*_(_ *_D_*_)_, *E*_(_ *_D_*_)_), *N*_(_ *_D_*_)_ is the set of points that contain all the diseases in the DAG(*D*). *E*_(_ *_D_*_)_ is the set of edges that contain all relationships between nodes in the DAG(*D*). For diseases that are included in MeSH, the semantic similarity that is calculated by means of DAG can be chose to represent the disease according to the previous literature. The semantic similarity between different diseases can be defined as follows. In DAG of disease *D*, the contribution of any ancestral disease *t* to disease *D* is as the formula:

(2) $$D1_{D}(t)=\{\begin{array}{ll} 1 & ,if\;t=D\\ \max\left\{∆*D1_{D}(t^{\prime })|t'\in \;children\;of\;t,\right\} & ,if\;t\neq D \end{array}$$

Δ is the semantic contribution factor. The contribution of disease *D* to itself is 1 and the contribution of other nodes to *D* will be attenuated due to Δ. Based on equation (Barabási and Oltvai, 2004), we can obtain the sum of the contributions of all diseases in DAG to *D*:

(3) $$DV1(D)=\Sigma_{t\in N_{D}}D1_{D}(t)$$

Like the Jaccard similarity coefficient, the semantic similarity between the diseases *i* and *j* can be calculated by the following formula:

(4) $$S1(i,j)=\frac{\sum_{t\in N_{i}\cap^{\text{​}}N_{j}}\left(D1_{i}(t)+D1_{j}(t)\right)}{DV1(i)+DV1(j)}$$

Considering that the dimensions of the attribute feature vector of different kinds of nodes are not uniform, we trained a Deep Autoencoder (DAE) to learn its hidden high-level low-rank representation and unify its dimensions.

### Deep Autoencoder

For different kind of node attributes, their dimensions are not same. The DAE is used to learn the high-level hidden distribution of different types of attribute features to obtain uniform feature vectors for downstream machine learning task. The core ideas of DAE are briefly reviewed in this section for self-contained. It is an unsupervised deep learning model consisting of two parts: encoder and decoder. The encoder consists of several nonlinear functions that map the input data to the representation space. The decoder includes a plurality of non-linear functions that map representations in the representation space for the reconstruction space. For a given input *x*, DAE maps the input to the output *O*(*x*):

(5) $$O_{\left(w,b\right)}(x)=f(W^{T}x)=f\left(\sum_{i=1}^{n}w_{i}x_{i}+b\right)$$

where the nonlinear activation function *f* can be defined as:

(6) f(t)=max(0,Wt+b)

Suppose the output of *O*(*x*) is $\hat{x}$ DAE aims to minimize the error between input and output. The loss function can be defined as follow:

(7) $$ℒ=\overset{n}{\underset{i=1}{\sum }}{\left\|{\hat{x}}_{i}-x_{i}\right\|}_{2}^{2}$$

## Results and Discussion

### Performance Evaluation Indicators

In this study, the five-fold cross-validation was adopted to fairly evaluate the performance of this model. First, we will briefly introduce the scheme of five-fold cross validation. The entire data set is randomly divided into five equal parts, each taking four subsets as the training set and the remaining one subset as the test set, cycle five times in turn, take the average of five times as the final performance. The widely used evaluation measure is adopted to evaluate our method, including accuracy (Acc.), sensitivity (Sen.), also means recall, specificity (Spec.), precision (Prec.), and Matthews correlation coefficient (MCC), and they can define as:

(8) $$\text{Acc}.=\frac{TN+TP}{TN+TP+FN+FP}$$

(9) $$\text{Sen}.=\frac{TP}{TP+FN}$$

(10) $$\text{Spec}.\;=\frac{TN}{TN+FP}$$

(11) $$\text{Prec}.=\frac{TP}{TP+FP}$$

(12) $$\text{MCC}=\frac{TP\times TN-FP\times FN}{\sqrt{\left(TP+FP)(TP+FN)(TN+FP)(TN+FN\right)}}$$

where *TN* represents the correctly predicted number of negative samples, *TP* stands for the correctly predicted number of positive samples, *FN* indicates the wrongly predicted number of negative samples and *FP* denotes the wrongly predicted number of positive samples. Certainly, the receiver operating characteristic (ROC) curve, precision-recall curve and the area under the ROC curve (AUC), the area under the precision-recall curve (AUPR) is also adopted to evaluate the performance of MAN-HOPE.

### Performance of Association Prediction Between Any Two Molecules

**Figure 4:**
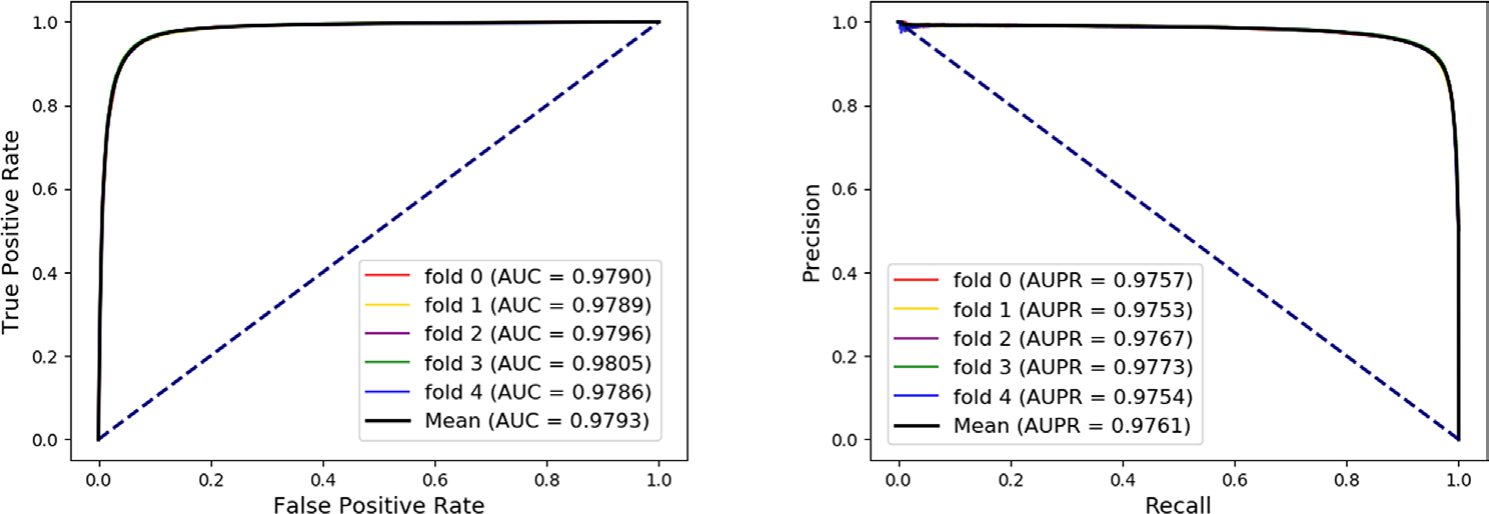
The five-fold cross validation receiver operating characteristic (ROC), precision-recall curve, area under the ROC curve (AUC), and area under the precision-recall curve of Molecular Association Network (MAN)–High-Order Proximity preserved Embedding (HOPE) on the entire MAN dataset.

In order to evaluate the performance of our method, the five-fold cross-validation was adopted. For MAN, we remove 20% of the links each time as the training set, the removed links is the testing set, and ensure that the links removed in these five times have no overlap. In each fold cross validation, we only use the training set as input to the network representation learning model to learn the behavior (network embedding) feature of nodes. Our model can predict any association between nodes in the MAN. The five-fold cross validation performance is shown in **Table 1** and **Figure 4** as follows.

**Table 1:** The five-fold cross-validation performance of MAN-HOPE on MAN dataset.

Fold	Acc. (%)	Sen. (%)	Spec. (%)	Prec. (%)	MCC (%)	AUC (%)
0	93.29	91.49	95.09	94.91	86.64	97.90
1	93.21	91.40	95.02	94.83	86.48	97.89
2	93.22	91.40	95.05	94.86	86.50	97.96
3	93.51	91.73	95.28	95.11	87.07	98.05
4	93.27	91.50	95.04	94.86	86.60	97.86
**Average**	**93.30 ± 0.12**	**91.50 ± 0.14**	**95.10 ± 0.11**	**94.91 ± 0.11**	**86.66 ± 0.24**	**97.93 ± 0.08**

The boldface indicates this measure performance is the best in this item among the compared methods.

On entire MAN, for predicting any type of molecular associations, that is, for predicting any link or edge in the association network, our method MAN-HOPE achieves an average accuracy of 93.30%, a sensitivity of 91.50%, a specificity of 95.10%, a precision of 94.91%, a MCC of 86.66%, an AUC of 97.93%, and an AUPR of 0.9761. It should be noted that our classifier only uses the default parameters and does not perform any parameter optimization. To characterize the volatility of the model's performance, we also calculated the standard deviation of the five-fold cross-validation. As can be seen from **Table 1**, the standard deviation of the above indicators is 0.12, 0.14, 0.11, 0.11, 0.24, and 0.08, which can reflect that our model MAN-HOPE is very stable and robust.

### Evaluate the Performance of Behavior and Attribute Features

**Figure 5:**
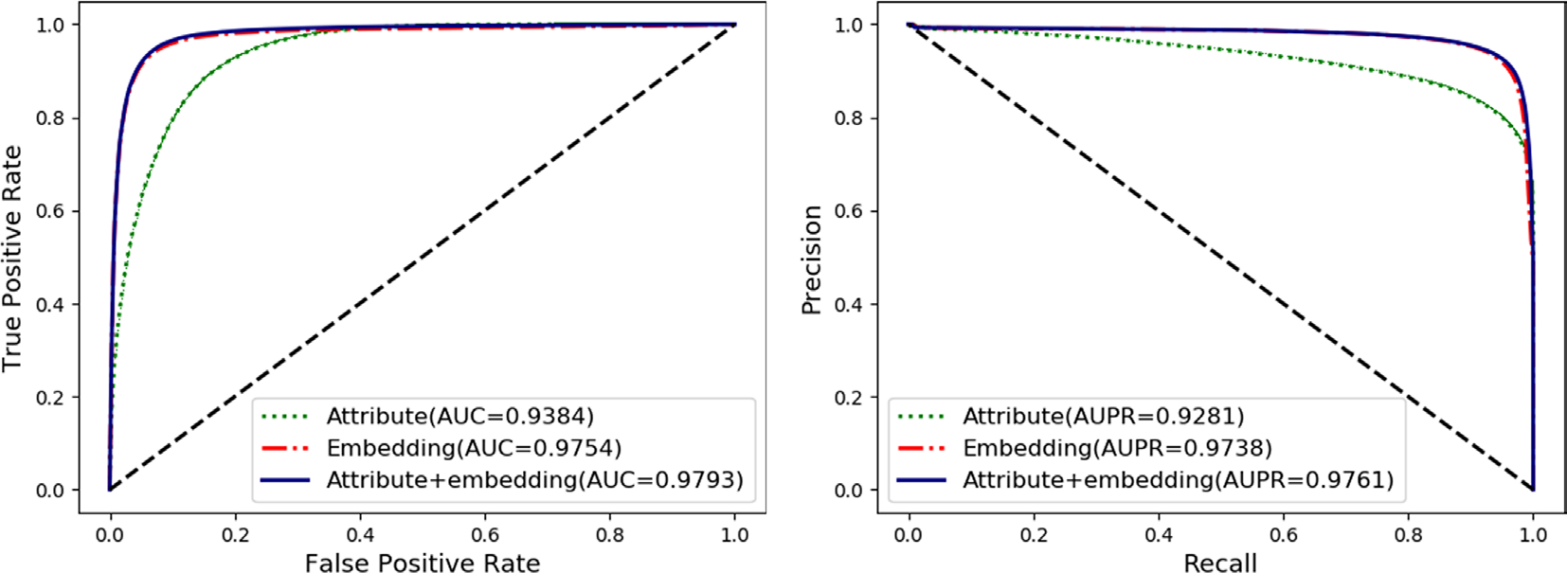
Comparison of nodes network embedding and attribute features.

To fully exploit the discriminative features of nodes, we considered both the behavior (network embedding) and the attribute of nodes. In this section, we will evaluate and compare the effects of individual behavior and attribute features and their combined use. The details are shown in **Table 2** and **Figure 5** as follow.

**Table 2:** Comparison of different features.

Feature	Acc. (%)	Sen. (%)	Spec. (%)	Prec. (%)	MCC (%)	AUC (%)
Attribute	86.52 ± 0.24	**92.08 ± 0.30**	80.96 ± 0.24	82.87 ± 0.21	73.50 ± 0.49	93.84 ± 0.18
Behavior	92.93 ± 0.11	90.56 ± 0.10	**95.29 ± 0.12**	**95.06 ± 0.12**	85.95 ± 0.22	97.55 ± 0.08
Combined use	**93.30 ± 0.12**	91.50 ± 0.14	95.10 ± 0.11	94.91 ± 0.11	**86.66 ± 0.24**	**97.93 ± 0.08**

The boldface indicates this measure performance is the best in this item among the compared methods.

The attribute feature of each type of node is obtained by the most widely used feature extraction methods in its related research, such as k-mer for lncRNA, miRNA, and protein sequences, fingerprints for drug chemical structure, and semantic similarity for the disease phenotype. The behavior features of nodes are learned by the HOPE algorithm on the training set. In order to evaluate the impact of each kind of feature on the final classification performance of the MAN-HOPE model, we executed our model separately using individual behavior feature, attribute feature, and combined use of these two features. As the results listed in **Table 2** and **Figure 5**, the performances of behavior feature are significantly better than those of attribute features. Moreover, behavior feature has contributed to the final performance, and attribute feature have also played a role in performance improvement.

### Comparison of Widely Used Machine Learning Models

**Figure 6:**
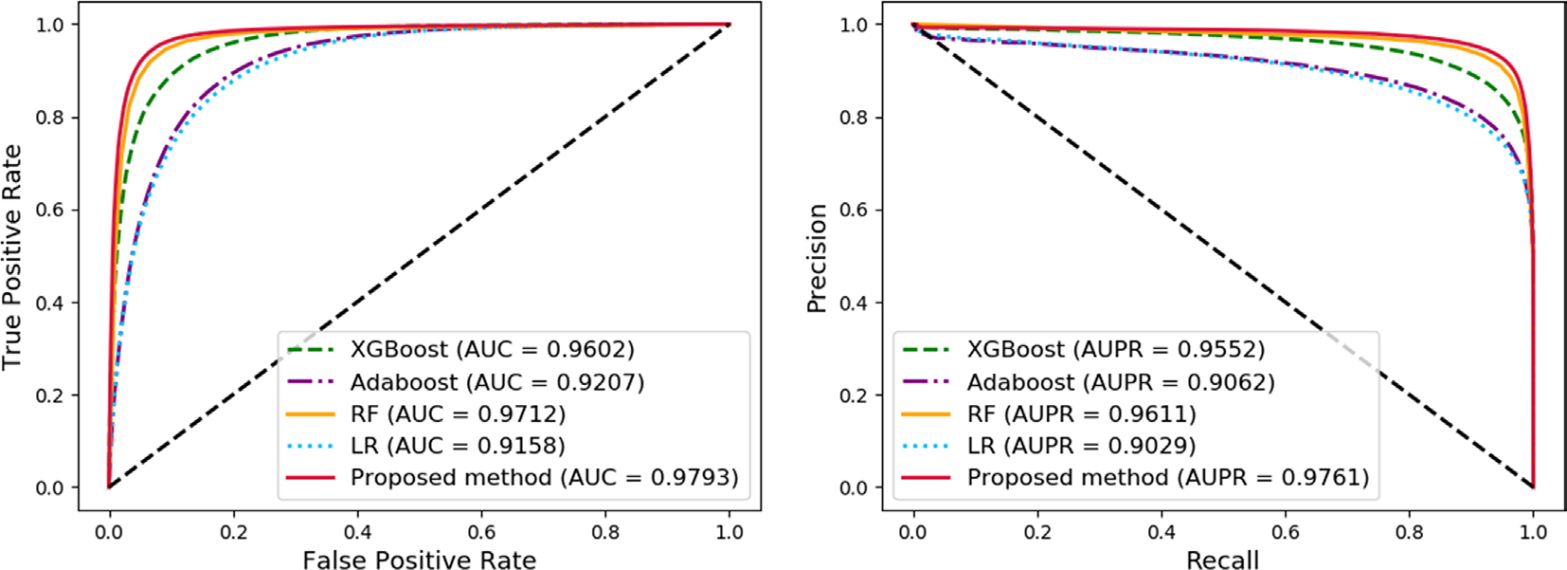
Comparison of widely used machine learning models and Molecular Association Network (MAN)–High-Order Proximity preserved Embedding (HOPE).

In order to compare the performance of our method, we compared the proposed method with other widely used machine learning models, including Logistic Regression (LR), AdaBoost, Random Forest (RF), and XGBoost, under the same experimental conditions, named as MAN-HOPE-LR, MAN-HOPE-Ada, MAN-HOPE-RF, and MAN-HOPE-XGB, respectively. Both the proposed method and other contrast models use only default parameters to avoid bias. The results are shown in **Table 3** and **Figure 6** as below.

**Table 3:** Compare with widely used machine learning models.

Method	Acc. (%)	Sen. (%)	Spec. (%)	Prec. (%)	MCC (%)	AUC (%)
MAN-HOPE-LR	83.75 ± 0.11	83.21 ± 0.47	84.30 ± 0.32	84.13 ± 0.20	67.52 ± 0.22	91.58 ± 0.13
MAN-HOPE-Ada	84.73 ± 0.18	85.53 ± 0.29	83.93 ± 0.22	84.19 ± 0.18	69.48 ± 0.36	92.07 ± 0.13
MAN-HOPE-RF	92.66 ± 0.12	**92.03** **±** **0.15**	93.29 ± 0.22	93.21 ± 0.20	85.33 ± 0.24	97.12 ± 0.05
MAN-HOPE-XGB	89.56 ± 0.41	90.60 ± 0.28	88.51 ± 0.95	88.75 ± 0.81	79.13 ± 0.79	96.02 ± 0.24
**Proposed method**	**93.30 ± 0.12**	91.50 ± 0.14	**95.10 ± 0.11**	**94.91 ± 0.11**	**86.66 ± 0.24**	**97.93 ± 0.08**

The boldface indicates this measure performance is the best in this item among the compared methods.

The proposed method achieves the best results with a high accuracy of 93.30% and a remarkable AUC of 97.93, while also having the smallest standard deviation. LR is a baseline model for many machine learning applications and usually has a relatively stable performance. AdaBoost is a weak classifier integrated algorithm that perform well in many tasks. RF is a decision tree-based algorithm with strong performance and interpretability. XGBoost is also an implementation of the GBDT algorithm. The results of the comparative experiments demonstrate the superior performance of our framework.

### Case Study: Predicting miRNA-Disease Associations

**Figure 7:**
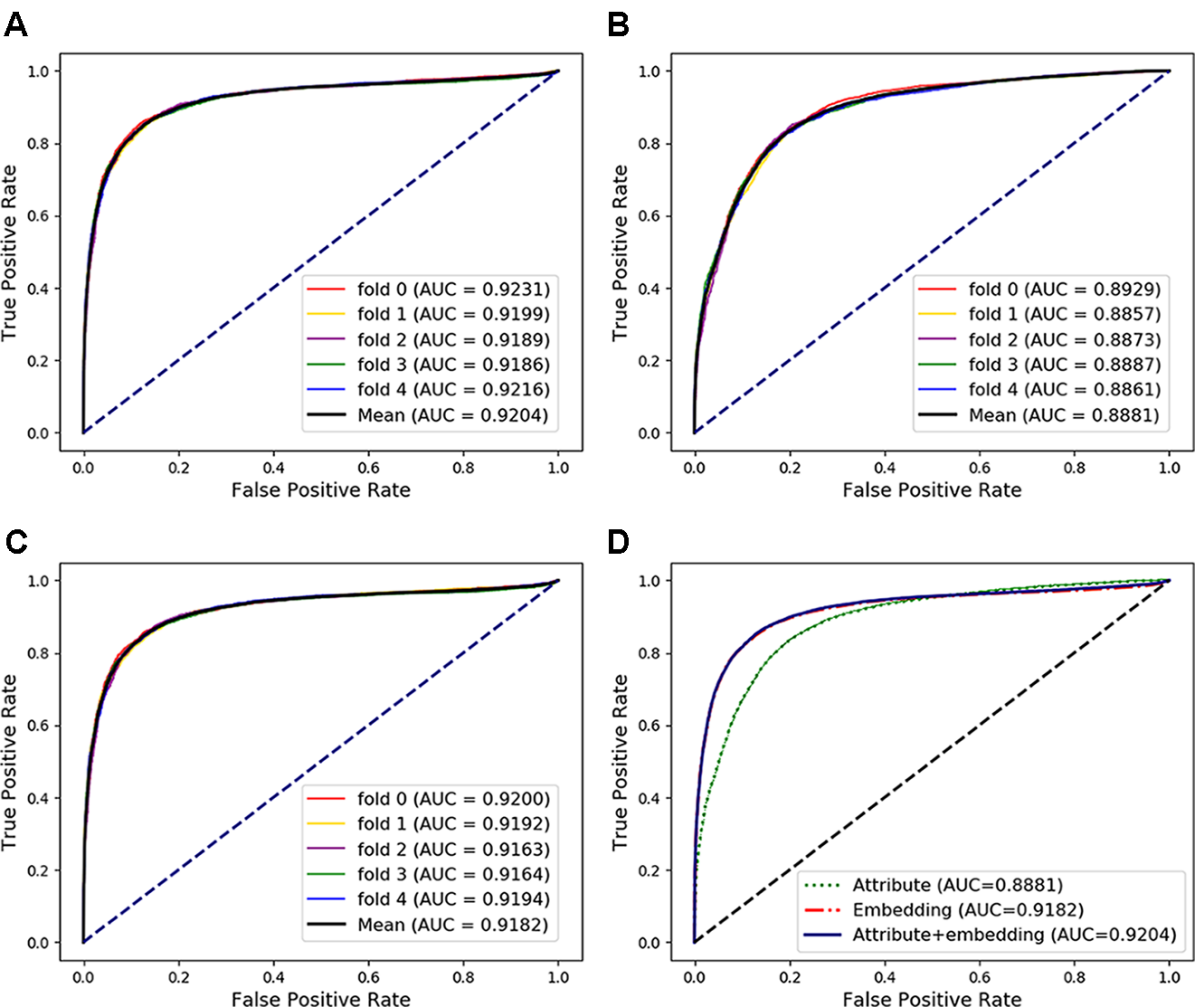
The performance of Molecular Association Network (MAN)–High-Order Proximity preserved Embedding (HOPE) for predicting miRNA-disease associations. **(A)** five-fold cross-validation performance using behavior (network embedding) feature and attribute feature of nodes; **(B)** five-fold cross-validation performance using only attribute feature; **(C)** five-fold cross-validation performance using only behavior feature; **(D)** comparison of performance of attribute feature, behavior feature, and combined use for predicting miRNA-disease associations.

In the above experiments, the capability of our method to predict arbitrary interactions has been verified. In this section, to verify the prediction ability of specific types of associations, the MAN-HOPE was used to predict miRNA-disease associations. We divided the miRNA-disease links in the whole MAN into five equal subsets, take four subsets each time as a training set and the remaining one subset as the testing set. The training set and all remaining associations in the MAN are used to learn behavior embedding feature for miRNA and disease nodes (remove the testing set from the whole MAN). The *k*-mer and semantic similarity of miRNA and disease are used as node attribute features. Results of MAN-HOPE for predicting miRNA-disease associations are shown in **Table 4** and **Figure 7**. As shown in **Figure 7A**, MAN-HOPE can achieve good performance with an accuracy of 85.89% and an AUC of 92.04%. The effects of attribute, behavior feature for predicting miRNA-disease associations were also compared. The performances are shown in **Figures 7B–D**. The results indicate the prediction ability of MAN-HOPE to predict any association between any type of molecules in the MAN.

**Table 4:** The five-fold cross-validation performance of MAN-HOPE for predicting miRNA-disease associations.

Fold	Acc. (%)	Sen. (%)	Spec. (%)	Prec. (%)	MCC (%)	AUC (%)
0	86.32	82.14	90.51	89.64	72.9	92.14
1	85.48	81.65	89.32	88.43	71.18	92.17
2	85.86	81.53	90.2	89.27	72	92.04
3	85.56	80.74	90.38	89.36	71.45	91.73
4	86.23	82.3	90.16	89.32	72.69	92.13
**Average**	**85.89 ± 0.38**	**81.67 ± 0.61**	**90.11 ± 0.47**	**89.20 ± 0.46**	**72.04 ± 0.75**	**92.04 ± 0.18**

The boldface indicates this measure performance is the best in this item among the compared methods.

## Conclusion

In this study, we construct a molecular association network by integrating various intermolecular associations in human based on a systematic view. We present a network-based model, MAN-HOPE, in which any association between molecules can be predicted. Network representation learning algorithm HOPE is applied to learn behavior feature of nodes. And the attribute feature of nodes, e.g. the k-mer frequency of sequence, fingerprint of chemical structure, and semantic similarity of disease phenotype is also adopted. The GBDT classifier implemented by CatBoost is trained to predict the associations between molecules. And the effectiveness and robustness of our framework are verified by rigorous experiments. In addition, we did a case study using MAN-HOPE to predict miRNA-disease associations, which indicate the ability of the proposed method for predicting specific type of associations in the entire network.

## Data Availability Statement

The data that support the findings of this study are available at https://github.com/haichengyi/MAN-V1

## Author Contributions

H-CY conceived the algorithm, carried out analyses, prepared the data sets, carried out experiments, and wrote the manuscript; Z-HY and Z-HG designed, performed and analyzed experiments and wrote the manuscript; All authors read and approved the ﬁnal manuscript.

## Funding

This work is supported by the National Natural Science Foundation of China, under Grant 61572506, in part by the National Outstanding Youth Science Fund Project of National Natural Science Foundation of China, under Grant 61722212, in part by the Pioneer Hundred Talents Program of Chinese Academy of Sciences.

## Conflict of Interest

The authors declare that the research was conducted in the absence of any commercial or financial relationships that could be construed as a potential conflict of interest.
